# Distinct T-helper cell responses to *Staphylococcus aureus* bacteremia reflect immunologic comorbidities and correlate with mortality

**DOI:** 10.1186/s13054-018-2025-x

**Published:** 2018-04-25

**Authors:** Jared A. Greenberg, Cara L. Hrusch, Mohammad R. Jaffery, Michael Z. David, Robert S. Daum, Jesse B. Hall, John P. Kress, Anne I. Sperling, Philip A. Verhoef

**Affiliations:** 1 Division of Pulmonary and Critical Care Medicine, Department of Medicine, Rush University Medical Center, 1725 West Harrison Street, Suite 054, Chicago, IL 60612 USA; 2 Section of Pulmonary and Critical Care Medicine, Department of Medicine, University of Chicago, Chicago, IL USA; 3 Division of Infectious Disease, Department of Medicine, University of Pennsylvania, Philadelphia, PA USA; 4 Section of Infectious Disease and Global Health, Department of Pediatrics, University of Chicago, Chicago, IL USA; 5 Committee on Immunology, University of Chicago, Chicago, IL USA

**Keywords:** Sepsis, *Staphylococcus aureus*, Helper T cells

## Abstract

**Background:**

The dysregulated host immune response that defines sepsis varies as a function of both the immune status of the host and the distinct nature of the pathogen. The degree to which immunocompromising comorbidities or immunosuppressive medications affect the immune response to infection is poorly understood because these patients are often excluded from studies about septic immunity. The objectives of this study were to determine the immune response to a single pathogen (*Staphylococcus aureus*) among a diverse case mix of patients and to determine whether comorbidities affect immune and clinical outcomes.

**Methods:**

Blood samples were drawn from 95 adult inpatients at multiple time points after the first positive *S. aureus* blood culture. Cox proportional hazards modeling was used to determine the associations between admission neutrophil counts, admission lymphocyte counts, cytokine levels, and 90-day mortality. A nested case-control flow cytometric analysis was conducted to determine T-helper type 1 (Th1), Th2, Th17, and regulatory T-cell (Treg) subsets among a subgroup of 28 patients. In a secondary analysis, we categorized patients as either having immunocompromising disorders (human immunodeficiency virus and hematologic malignancies), receiving immunosuppressive medications, or being not immunocompromised.

**Results:**

Higher neutrophil-to-lymphocyte count ratios and higher Th17 cytokine responses relative to Th1 cytokine responses early after infection were independently associated with mortality and did not depend on the immune state of the patient (HR 1.93, 95% CI 1.17–3.17, *p* = 0.01; and HR 1.13, 95% CI 1.01–1.27, *p* = 0.03, respectively). On the basis of flow cytometric analysis of CD4 T-helper subsets, an increasing Th17/Treg response over the course of the infection was most strongly associated with increased mortality (HR 4.41, 95% CI 1.69–11.5, *p* < 0.01). This type of immune response was most common among patients who were not immunocompromised. In contrast, among immunocompromised patients who died, a decreasing Th1/Treg response was most common.

**Conclusions:**

The association of both increased Th17 responses and increased neutrophil counts relative to lymphocyte counts with mortality suggests that an overwhelming inflammatory response is detrimental. However, the differential responses of patients according to immune state suggest that immune status is an important clinical indicator that should be accounted for in the management of septic patients, as well as in the development of novel immunomodulatory therapies.

**Electronic supplementary material:**

The online version of this article (10.1186/s13054-018-2025-x) contains supplementary material, which is available to authorized users.

## Background

Sepsis is a leading cause of death among critically ill patients [1]. The current paradigm of sepsis pathophysiology suggests that some patients will die early in the disease course as a result of a dysregulated proinflammatory phase (systemic inflammatory response syndrome [SIRS]). However, many more patients will die later of subsequent insults during a predominating immunosuppressive recovery phase (compensatory anti-inflammatory response syndrome [CARS]) [2]. The SIRS response is characterized by high levels of interleukin-1β (IL-1β), tumor necrosis factor-α (TNF-α), and expression of human leukocyte antigen (HLA)-DR on monocytes. In contrast, anti-inflammatory IL-10 and reduced expression of HLA-DR on monocytes are characteristics of the CARS response.

Recently, investigators have observed that certain septic patients never mount a clinically evident SIRS response, suggesting that the presence of SIRS should not be considered an essential component of the sepsis clinical syndrome [3]. The reason that some patients with infections and associated organ failure do not exhibit a SIRS response may be that preexisting chronic conditions influence the nature of the SIRS and CARS responses during the course of infection [4]. Studies of the septic immune response often exclude patients with disorders of the immune system or patients undergoing treatments that reduce immunity [5–8]. As a result, the septic immune response is not well characterized for at least 25% of patients with sepsis [9]. Moreover, any immune response identified as beneficial or harmful in these studies may not be generalizable, owing to the exclusion of patients with immunocompromising comorbidities.

One of the main pathogens responsible for sepsis is *Staphylococcus aureus.* Prior studies of immune dysregulation in sepsis due to *S. aureus* infection were focused on the innate immune response, particularly TNF-α and IL-10 [10, 11]. These cytokines, along with IL-6, have long been known to be prognostic in different populations with sepsis. However, numerous attempts to block these cytokines have not led to improved outcomes [12]. Notably, monocytes isolated from septic patients that express higher levels of HLA-DR also produce more TNF-α after lipopolysaccharide stimulation [13]. In addition, polymorphisms in HLA-DR increase risk for *S. aureus* susceptibility, indicating that antigen presentation by monocytes to T cells may be a critical driver of disease outcome [14]. However, little is known about the resulting T-cell responses in patients with *S. aureus* bacteremia. One recent study indicated that T-helper type 1 (Th1) cells are expanded in immunocompetent patients and are protective in a murine model of infection [15]. In murine models, T-cell polarization during *S. aureus* infection is dependent on the route of administration, the dose of bacteria delivered, and postinfection day of analysis, making these studies difficult to translate to patients with naturally acquired bacteremia [16–21]. Interestingly, whereas Th17 cells have been implicated in protection in murine models of *S. aureus* infection, the role of Th17 cells in humans has not been established. Finally, the presence of *S. aureus*-specific antibodies does not correlate with resistance to infection [22]. Therefore, a protective memory T-cell response may be critical in patients with sepsis by maintaining a proper SIRS/CARS balance.

In the present study, we focused on the associations between differentially polarized immune responses after *S. aureus* bloodstream infection and 90-day mortality in a prospectively enrolled patient cohort. This approach allowed for evaluation of the host immune response independent of pathogen or pathogen-associated molecular pattern variability. We previously found that over 30% of patients with *S. aureus* bacteremia had either immunocompromising hematopoietic disorders (hematologic malignancies or human immunodeficiency virus [HIV] infection) or received medications that interfered with immune function for the management of solid malignancies, solid organ transplants, or rheumatologic conditions [23]. Thus, our secondary goal was to explore whether the immune responses associated with mortality after *S. aureus* bacteremia occurred more or less frequently among these patient groups that are often excluded from studies of the septic immune response.

## Methods

### Sample collection

This study was conducted at the University of Chicago Medical Center (Chicago, IL, USA), a 547-bed, university-affiliated urban teaching hospital, between July 1, 2013, and October 24, 2014. The University of Chicago Institutional Review Board approved this study. All adult inpatients with at least one positive blood culture for *S. aureus* within the previous 4 days were approached for participation. Informed consent was obtained by the patients or their surrogates.

Blood samples were drawn into ethylenediaminetetraacetic acid tubes at three distinct time points (2–4 days, 6–9 days, and 12–18 days after the first day of positive *S. aureus* blood cultures). It was necessary to include day ranges within time points, because there was variability in the time it took blood cultures to turn positive and we were unable to process samples every day of the week. The first two time points were chosen on the basis of previous sepsis biomarker studies [24, 25], and we added a third time point to strengthen our analysis. Within 2 hours of collection, plasma and peripheral blood mononuclear cells (PBMCs) were isolated via differential centrifugation over Histopaque-1077 (Sigma-Aldrich, St. Louis, MO, USA). The samples were cryopreserved until analysis. Other investigators have reported consistent measurements when performing flow cytometry on fresh vs. thawed cells [26, 27]. Clinical and laboratory data (including complete blood count with differential) were abstracted from the patients’ medical records.

### Cytokine analysis

A multiplex assay (EMD Millipore, Darmstadt, Germany) was used to determine plasma cytokine concentrations at the first two time points (days 2–4 and 6–9). Cytokine levels that were below the limit of detection were assigned the lowest extrapolated value for each cytokine. Cytokine concentrations were log-transformed and were treated as continuous variables.

### Flow cytometry

Thawed PBMCs were washed twice in fluorescence-activated cell sorting buffer (PBS containing 0.1% sodium azide and 0.2% bovine serum albumin) and incubated with a viability dye for 15 minutes (Zombie Aqua, BioLegend, San Diego, CA, USA). Cells were incubated for 10 minutes with pooled human immunoglobulin G to block nonspecific antibody binding (FcX solution; BioLegend), and surface staining was performed using fluorescently conjugated antibodies CD3-fluorescein isothiocyanate, CD4-allophycocyanin (APC), CCR4-phycoerythrin (PE), CCR6-Brilliant Violet 605, CD45RO-Brilliant Violet 711, CD25-APC/cyanine 7 (Cy7), and CD127-PE/Cy7 (BioLegend). Flow cytometry data were acquired on an LSRFortessa (BD Biosciences, San Jose, CA, USA) and analyzed with FlowJo software (FlowJo, Ashland, OR, USA).

### Statistical analysis

The primary outcome was death during the first 90 days after bacteremia, which was determined by reviewing the medical record or contacting the patient’s surrogate. Our planned enrollment was 90 patients, of which we expected at least 20 to die during follow-up. On the basis of this enrollment target, our study was powered to include at least two immune markers in a multivariable model. In our secondary analysis, we explored whether the immune responses associated with mortality occurred more or less frequently among the patients with immunocompromising conditions or taking immunosuppressive medications.

The *t* test, Mann-Whitney *U* test, χ^2^ test, or Fisher’s exact test was used in bivariate testing, as appropriate, to determine if differences between groups were significant. In our primary analysis, we determined the associations between immune marker levels and 90-day mortality using Cox proportional hazards modeling. In multivariable models, we removed variables that were not statistically significant using backward selection (*p* > 0.05). In our secondary analysis, where outcomes were trends in cellular immune markers over time, we used linear mixed models to account for correlated measurements from the same patient [28]. We determined that a linear model approximated these relationships because an additional “time after infection squared” term did not reach statistical significance (*p* > 0.05). All tests were two-sided. All analyses were performed with STATA 13.1 software (StataCorp, College Station, TX, USA).

## Results

### Neutrophil-to-lymphocyte count ratio on day of infection was associated with increased mortality after *S. aureus* bacteremia

Of the 153 patients considered for enrollment, 95 (62%) were enrolled (Additional file 1: Figure S1). There were 21 patients (22%) who died during the 90 days after *S. aureus* infection; there were 16 deaths that occurred during the first 30 days and 5 deaths that occurred during days 31–90. Bivariate unadjusted analysis revealed that patients who died were more likely to be older, to have higher Sequential Organ Failure Assessment (SOFA) scores, to meet SIRS heart rate criteria, and to have a pulmonary source of infection than patients who survived (Table 1)*.* We did not find significant associations between methicillin resistance or the duration of bacteremia and survival.

**Table 1:** Baseline characteristics of 95 patients with *Staphylococcus aureus* bacteremia

		Survived (*n* = 74)	Died (*n* = 21)	*p* Value for difference
Patient demographics	Age, years, mean [SD]	56 [16]	65 [13]	0.02
	Male sex, *n* (%)	46 (62)	12 (57)	0.68
	Black race, *n* (%)	47 (64)	12 (57)	0.60
	Solid malignancy^a^, *n* (%)	12 (16)	7 (33)	0.12
	Hematological malignancy, *n* (%)	7 (9)	0 (0)	0.34
	Autoimmune condition^a^, *n* (%)	8 (11)	4 (19)	0.46
	Solid organ transplant^a^, *n* (%)	4 (5)	0 (0)	0.57
	HIV infection, *n* (%)	2 (3)	0 (0)	1.00
	Diabetes, *n* (%)	26 (35)	8 (38)	0.80
	Congestive heart failure, *n* (%)	21 (28)	8 (38)	0.43
	End-stage renal disease, *n* (%)	20 (27)	4 (19)	0.58
	Coronary artery disease, *n* (%)	16 (22)	5 (24)	0.78
Clinical response to infection	Total WBC^b^ on first day of bacteremia, 1000 cells/μl, mean [SD]	13 [7]	17 [10]	0.37
	Neutrophils^b^ on first day of bacteremia, 1000 cells/μl, mean [SD]	11 [6.0]	14 [8]	0.35
	Lymphocytes^b^ on first day of bacteremia, 1000 cells/μl, mean [SD]	1.0 [0.8]	0.7 [0.7]	0.22
	Neutrophils/lymphocytes^b^ on first day of bacteremia, mean [SD]	15 [16]	27 [21]	0.01
	Positive SIRS temperature criteria, *n* (%)	57 (77)	12 (57)	0.07
	Positive SIRS heart rate criteria, *n* (%)	51 (69)	21 (100)	0.003
	Positive SIRS respiratory criteria, *n* (%)	68 (92)	18 (86)	0.40
	Positive SIRS WBC criteria, *n* (%)	53 (71)	17 (81)	0.40
	SOFA score on first day of positive blood cultures, mean [SD]	3 [2]	6 [4]	0.001
	Time to antibiotics, h, median (IQR)	2.0 (0.8–8.9)	3.0 (1.0–5.8)	0.98
Characteristics of Infection	Methicillin resistance, *n* (%)	28 (38)	7 (24)	0.30
	Positive cultures < 48 h from hospital admission, *n* (%)	65 (89)	17 (81)	0.46
	Days of consecutive positive blood cultures, mean [SD]	3 [3]	3 [2]	0.93
	Endocarditis, *n* (%)	6 (8)	4 (19)	0.23
	Removable source^c^, *n* (%)	26 (35)	7 (33)	1.0
	Skin/soft tissue, *n* (%)	23 (31)	3 (14)	0.17
	Pulmonary source, *n* (%)	6 (8)	6 (29)	0.02
	Other source, *n* (%)	5 (7)	0 (0)	0.58
	Undetermined source, *n* (%)	14 (19)	5 (24)	0.76

*Abbreviations: SIRS* Systemic inflammatory response syndrome, *HIV* Human immunodeficiency virus, *WBC* White blood cells, *SOFA* Sequential Organ Failure Assessment

Patients were grouped as those who had died or survived at day 90 after bacteremia

^a^Patients with these conditions were receiving chemotherapy or immune-modulating medications

^b^Values were log-transformed prior to performing statistical tests

^c^Examples of removable sources are intravenous lines and pacemaker wires

**Fig. 1:**
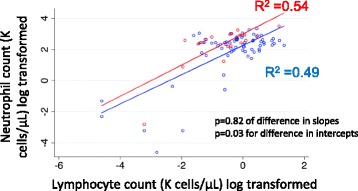
Association between neutrophil and lymphocyte counts on the first day of *Staphylococcus aureus* bacteremia. Patients were categorized as non-survivors (*red*) or survivors (*blue*). Linear regression lines were generated for both survivors and non-survivors

We also examined the neutrophil-to-lymphocyte count ratio among survivors and non-survivors, because a higher level has been cited as a negative prognostic factor in studies of sepsis and critical illness [29, 30]. Whereas the absolute neutrophil and lymphocyte counts on the first day of bacteremia were similar among survivors and non-survivors, patients who died had significantly higher neutrophil-to-lymphocyte count ratios than patients who survived (27 vs. 15, *p* = 0.01 for difference). We created a scatterplot with each patient’s neutrophil count on the *y*-axis and each patient’s lymphocyte count on the *x*-axis (Fig. 1). Linear regression lines were created for survivors and for non-survivors. The intercept of the regression line for non-survivors was significantly greater than the intercept for survivors (*p* = 0.03 for difference). The slopes were similar for survivors and non-survivors (*p* = 0.82 for difference). Thus, on average, patients who died had higher neutrophil counts relative to lymphocyte counts than patients who survived, suggesting that early differences in the immune response may predict long-term outcomes.

### High Th17 and low Th1 pathway cytokine profiles were associated with mortality independent of neutrophil and lymphocyte counts

IL-6 and IL-17A were the primary cytokines positively and significantly associated with mortality at time point 1 (2–4 days after infection) (HR 1.72, 95% CI 1.25–2.37, *p* = 0.001) and (HR 2.36, 95% CI 1.35–4.15, *p* = 0.003), respectively (Additional file 2: Table S1). IL-6 was the only cytokine positively and significantly associated with mortality at time point 2 (6–9 days after infection) (HR 2.03, 95% CI 1.31–3.14, *p* = 0.002) (Additional file 2: Table S2). Cytokines were grouped by immune pathway. For a particular pathway, we included not only cytokines produced by that type of T cell but also cytokines derived from innate cells that influenced T-cell differentiation into that pathway.

To evaluate the overall immune response for an individual patient and to address the statistical problem of multiple comparisons, we used a previously reported approach to combine cytokines common to specific pathways to generate Th17, Th1, and Th2 composite scores [31–33] (Additional file 2: Table S3). Each cytokine level was standardized by dividing its measured value by the cohort’s median value. Each patient’s Th17, Th1, and Th2 scores were determined by summing all log-standardized cytokine concentrations from the same immune pathway.

Using unadjusted Cox proportional hazards modeling, each of the Th17, Th1, and Th2 scores at days 2–4 after infection was not associated with death during the 90 days after infection (Table 2). However, using a multiple variable Cox proportional hazards model of Th17 and Th1 scores, a higher Th17 score was harmful (HR 1.18, 95% CI 1.05–1.31, *p* = 0.003), whereas a higher Th1 score was protective (HR 0.81, 95% CI 0.68–0.96, *p* = 0.02). Similarly, using a model of Th17 and Th2 scores, a higher Th17 score was harmful (HR 1.21, 95% CI 1.04–1.41, *p* = 0.01), whereas a higher Th2 score was protective (HR 0.86, 95% CI 0.74–1.00, *p* = 0.049). A model of Th1 and Th2 scores did not reveal any significant associations with mortality. There were similar associations when Th17, Th1, and Th2 scores measured at days 6–9 after infection were analyzed, but none reached statistical significance (Additional file 2: Table S4). Changes in Th17, Th1, and Th2 scores from time point 1 to time point 2 were not associated with mortality (Additional file 2: Table S5).

**Table 2:** Associations between Th17, Th1, and Th2 cytokine scores early after infection and death during first 90 days after *Staphylococcus aureus* bacteremia

	Associations between each variable and death during the first 90 days after infection			Multivariable model of Th17 and Th1 scores			Multivariable model of Th17 and Th2 scores			Multivariable model of Th1 and Th2 scores		
	HR	95% CI	*p* Value	HR	95% CI	*p* Value	HR	95% CI	*p* Value	HR	95% CI	*p* Value
Th17 score at time point 1	1.05	0.99–1.10	0.14	1.18	1.06–1.31	0.003	1.21	1.04–1.41	0.01	–	–	–
Th1 score at time point 1	0.98	0.89–1.08	0.66	0.81	0.68–0.96	0.02	–	–	–	0.88	0.73–1.06	0.17
Th2 score at time point 1	1.01	0.96–1.07	0.66	–	–	–	0.86	0.74–1.00	0.049	1.09	0.97–1.22	0.16

Associations between each helper T-cell (Th) score and death was determined using a Cox proportional hazards model. Three multivariable Cox proportional hazards models were performed, each including two of the three helper T cell scores as predictor variables. *Dashes* signify variables that were not included in the model

**Fig. 2:**
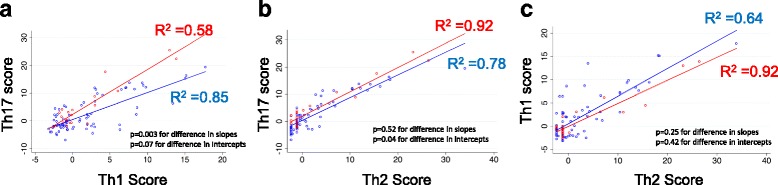
Associations between cytokine profile scores. Patients were categorized as non-survivors (*red*) or survivors (*blue*). Linear regression lines were generated for both survivors and non-survivors. Only values measured at time point 1 are displayed. **a** T-helper type 17 cell (Th17) vs. Th1 scores. **b** Th17 vs. Th2 scores. **c** Th1 vs. Th2 scores

We created scatterplots to examine the associations between Th17, Th1, and Th2 composite scores (Fig. 2). When each patient’s Th17 score was plotted against his or her Th1 score, we found that non-survivors had a significantly greater regression line slope than did survivors (*p* = 0.003 for difference) (Fig. 2a). This relationship illustrates why a high Th17 score was associated with increased risk of death only when it was high relative to the Th1 score. Similarly, when comparing the relationship between Th17 and Th2 scores, we found that non-survivors also had a significantly greater regression line intercept than did survivors (*p* = 0.04) (Fig. 2b). When comparing the relationship between Th1 and Th2 scores, we observed that the regression line slope and intercept were similar for survivors and non-survivors (Fig. 2c).

Finally, when we analyzed the neutrophil-to-lymphocyte count ratio, the Th17 score-to-Th1 score ratio, and the Th17 score-to-Th2 score ratio in a multivariable Cox proportional hazards model, we found that the neutrophil-to-lymphocyte count ratio and Th17 score-to-Th1 score ratio were independently associated with mortality (HR 1.93, 95% CI 1.17–3.17, *p* = 0.01; and HR 1.13, 95% CI 1.01–1.27, *p* = 0.03, respectively) (Table 3).

**Table 3:** Associations between neutrophils, lymphocytes, and helper T cell scores early after infection and death during the first 90 days after *Staphylococcus aureus* bacteremia

	Associations between each variable and death during first 90 days after infection			Multivariable model		
	HR	95% CI	*p* Value	HR	95% CI	*p* Value
Th17 score/Th1 score at time point 1	1.16	1.05–1.28	0.004	1.13	1.01–1.27	0.03
Th17 score/Th2 score at time point 1	1.20	1.03–1.40	0.02	NS	NS	NS
Neutrophils/lymphocytes on first day of bacteremia	1.94	1.18–3.2	0.009	1.93	1.17–3.17	0.01

Associations between each variable and death were determined using a Cox proportional hazards model. NS signifies a variable that was removed from the model for a *p* value > 0.05

### Neutrophil-to-lymphocyte count ratios and Th17 score-to-Th1 score ratios were similar, regardless of a patient’s immune state

Among the 95 patients in the cohort, there were 9 patients (9%) who had hematopoietic disorders (hematologic malignancies or HIV infections). None of these patients died during the 90-day follow-up period. There were 35 additional patients (37%) who received medications that interfered with immune function, including systemic steroids; 11 (31%) of these patients died during the 90-day follow-up period (Table 1). The clinical characteristics of patients grouped by the presence or absence of an immunocompromised state (either due to medication or due to disorder of hematopoietically derived cells) are displayed in Additional file 2: Table S6. Notably, patients with and without immunocompromising conditions were similar with respect to age and SOFA score, even though these patient characteristics were associated with mortality.

**Fig. 3:**
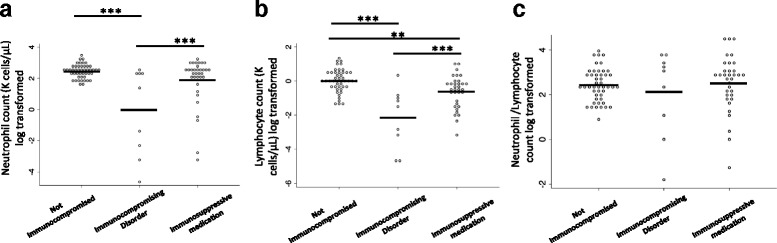
Neutrophil and lymphocyte counts for patients grouped by presence or absence of immunocompromising medical conditions or medications. **a** Neutrophil counts. **b** Lymphocyte counts. **c** Neutrophil-to-lymphocyte count ratio. *Solid lines* represent mean values. **p < 0.01, ***p < 0.001 by analysis of variance with Tukey’s posttest for multiple comparisons

**Fig. 4:**
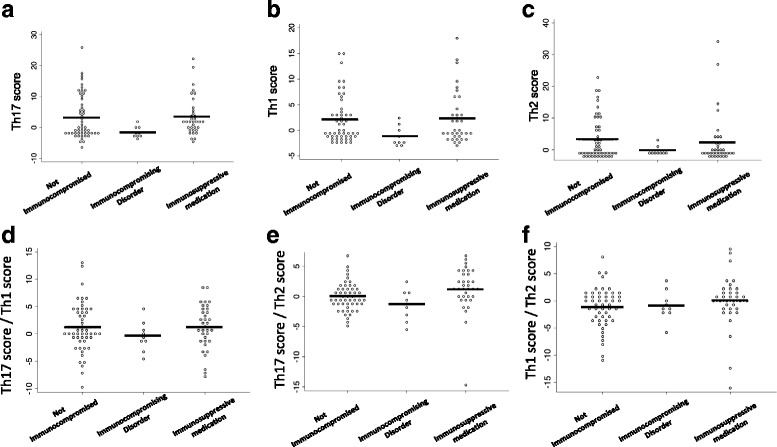
Cytokine profile scores for patients grouped by presence or absence of immunocompromising medical conditions or medications. Only values measured at time point 1 are displayed. **a** T-helper type 17 cell (Th17) score. **b** Th1 score. **c** Th2 score. **d** Th17 score/Th1 score. **e** Th17 score/Th2 score. **f** Th1 score/Th2 score. *Solid lines* represent mean values. *p* values were all > 0.05 by analysis of variance with Tukey’s posttest for multiple comparisons

Patients with hematopoietic disorders had lower neutrophil and lymphocyte counts than did patients who received immunosuppressive medications and patients who were not immunocompromised. However, the neutrophil-to-lymphocyte count ratio was similar across groups (Fig. 3a, b, c). The Th17, Th1, and Th2 composite scores were not significantly different between groups after *p* values were adjusted for multiple comparisons (Fig. 4a, b, c). Finally, the Th17/Th1, Th17/Th2, and Th1/Th2 score ratios were similar between groups (Fig. 4d, e, f). In summary, the immune marker ratios associated with survival (neutrophil-to-lymphocyte and Th17 score-to-Th1 score ratios) among patients typically labeled as immunocompromised were similar to those among patients without an immunocompromised state.

### Patients who died were more likely than patients who survived to have increased Th17 responses measured by flow cytometry

To determine if changes in T-helper cell differentiation were associated with death after *S. aureus* bacteremia, we performed a nested case-control flow cytometry study after we enrolled the first 67 patients in the cohort. There were 14 patients who died during the 90 days after bacteremia. Of these 14 patients, there were 7 who received immunosuppressive medications and 7 who did not. We selected a comparison group of 14 survivors with similar chronic medical problems and SOFA scores. There were no patients with hematologic malignancies or HIV infections who died, so there were no patients with these conditions evaluated in this aspect of the study. The clinical characteristics of the patients who were included and not included are displayed in Additional file 2: Table S7.

We used a previously validated flow cytometric approach to characterize T-cell subsets as Th1, Th2, Th17, or regulatory T cells (Tregs) [34, 35] (Additional file 3: Figure S2). Blood samples were drawn at days 2–4, 6–9, and 12–18 as long as the patient remained hospitalized. For *each* patient and for *each* T-cell subtype, we plotted the T-cell subtype percentage by the numbers of days after positive blood culture. Th1, Th2, and Th17 percentages were reported as the proportion of T cells exhibiting the surface markers corresponding to that subset of total effector CD45RO^+^CD4^+^ T cells. Tregs were reported as the proportion of CD25^hi^CD127^lo^ of total CD4 T cells. The trajectories of T-cell subsets for each patient are displayed in Additional file 4: Figure S3. Because Tregs are unique in their function to modulate the immune response, we also examined the ratios of Th1, Th2, and Th17 cells to Treg cells. We used linear regression to predict the change in each T-cell subset percentage per day for each patient. Each regression line was used to estimate a T-cell percentage at day 3 after infection (Additional file 5: Figure S4). The average values for these immune markers for survivors and non-survivors are displayed in Additional file 2: Table S8.

When these immune markers were analyzed in a multivariable Cox proportional hazards model, a high early Th2 response was associated with reduced mortality (HR 0.86, 95% CI 0.78–0.94, *p* < 0.01), whereas a high early Treg response was associated with increased mortality (HR 1.13, 95% CI 1.00–1.28, *p* = 0.046) (Table 4). Over the course of the infection, an increasing Th17/Treg response was highly associated with increased mortality (HR 4.41, 95% CI 1.69–11.5, *p* < 0.01).

**Table 4:** Associations between cellular markers and death during first 90 days after *Staphylococcus aureus* bacteremia

	Associations between each variable and death during first 90 days after infection			Multivariable model		
	HR	95% CI	*p* Value	HR	95% CI	*p* Value
Th17 at day 3	1.03	0.98–1.08	0.25	NS	NS	NS
Th1 at day 3	0.99	0.90–1.09	0.79	NS	NS	NS
Th2 at day 3	0.89	0.83–0.96	< 0.01	0.86	0.78–0.94	< 0.01
Treg at day 3	1.06	0.98–1.15	0.17	1.13	1.00–1.28	0.046
Th17/Treg at day 3	0.92	0.81–1.05	0.22	NS	NS	NS
Th1/Treg at day 3	0.70	0.45–1.09	0.11	NS	NS	NS
Th2/Treg at day 3	0.74	0.57–0.98	0.03	NS	NS	NS
ΔTh17/day	1.62	1.08–2.44	0.02	NS	NS	NS
ΔTh1/day	0.56	0.29–1.09	0.09	NS	NS	NS
ΔTh2/day	0.93	0.60–1.43	0.73	NS	NS	NS
ΔTreg/day	0.76	0.45–1.29	0.32	NS	NS	NS
Δ(Th17/Treg)/day	3.60	1.53–8.49	< 0.01	4.41	1.69–11.5	< 0.01
Δ(Th1/Treg)/day	7.0	0.17–283	0.30	NS	NS	NS
Δ(Th2/Treg)/day	3.0	0.51–17.2	0.22	NS	NS	NS

*Th* T-helper cell, *Treg* Regulatory T cell

Cellular markers measured early after infection and over the course of the infection were predictor variables. The association between each cellular marker and death was determined using a Cox proportional hazards model. A multivariable Cox proportional hazards model was used to determine the adjusted associations between cellular markers and death. Nonsignificant (NS) variables (*p* > 0.05) were removed from the multivariable model using backward selection

### Patients who did not receive immunosuppressive medications were more likely to have augmented Th17 responses than patients who did receive immunosuppressive medications

**Fig. 5:**
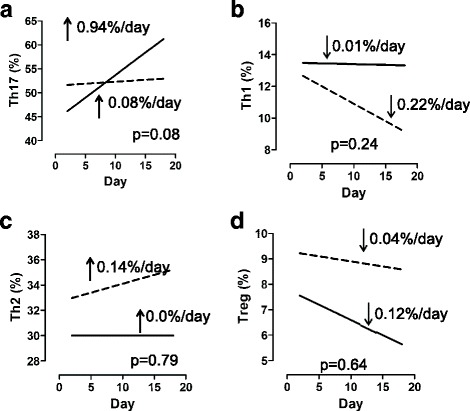
Trajectories of T-cell subsets. Average trends in immune markers were determined using linear mixed models. Patients who were not immunocompromised are represented by *solid lines*. Patients who were receiving immunosuppressive medications are represented by *dashed lines*. The *p* value is a test for differences in slopes between groups. **a** T-helper type 17 cells (Th17). **b** Th1 cells. **c** Th2 cells. **d** Regulatory T cells (Treg)

Using linear mixed models, we found that, on average, there were no differences in Th17, Th1, Th2, or Treg responses between our two patient groups (Fig. 5a–d). However, we did find a trend with Th17 responses, because Th17 cells increased by 0.94% per day among patients who were not receiving immunosuppressive medications and only by 0.08% per day among patients who were receiving immunosuppressive medications (*p* = 0.08 for difference) (Fig. 5a).

**Fig. 6:**
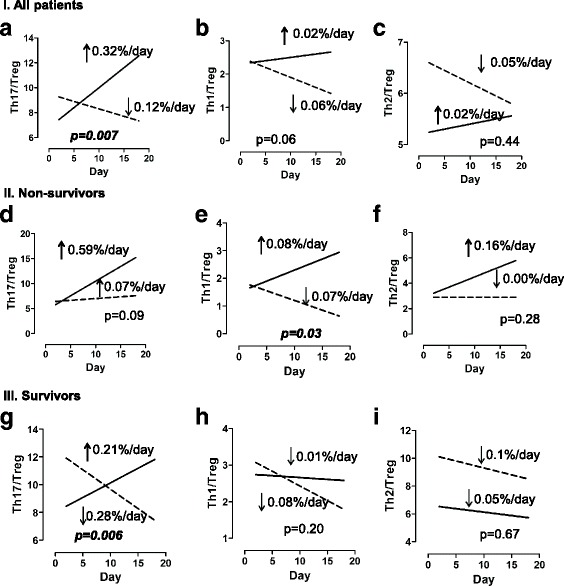
Trajectories of T-helper type 17 cell (Th17)/regulatory T cell (Treg), Th1/Treg, and Th2/Treg ratios. Average trends in immune markers were determined using linear mixed models. Patients who were not immunocompromised are represented by *solid lines*. Patients who were receiving immunosuppressive medications are represented by *dashed lines*. The *p* value is a test for differences in slopes between groups. **a**, **b**, **c** All 28 patients. **d**, **e**, **f** Only the 14 patients who died. **g**, **h**, **i** Only the 14 patients who survived

Although our investigation of each cell type individually led to no significant differences, our analysis of the degree of proinflammatory Th1, Th2, and Th17 responses relative to the anti-inflammatory Treg response revealed several findings. Strikingly, the Th17/Treg ratio *increased* by 0.32 per day among patients who were not receiving immunosuppressive medications and *decreased* by 0.12 per day among patients who were receiving immunosuppressive medications (*p* = 0.007 for difference) (Fig. 6a). Similarly, the Th1/Treg ratio *increased* by 0.02 per day among patients who were not receiving immunosuppressive medications and *decreased* by 0.06 per day among patients who were receiving immunosuppressive medications (*p* = 0.06 for difference) (Fig. 6b). Patients who were and those who were not receiving immunosuppressive medications had similar Th2/Treg trends (Fig. 6c).

When we considered only the 14 patients who died, we noted that patients who were not receiving immunosuppressive medications tended to die with increasing Th17/Treg ratios (*p* = 0.09 for difference) (Fig. 6d), whereas patients who were receiving immunosuppressive medications died with decreasing Th1/Treg ratios (*p* = 0.03 for difference) (Fig. 6e). The Th2/Treg trends were not associated with receiving immunosuppressive medications among the 14 patients who died (Fig. 6f). When we considered only the 14 patients who survived, we found that patients who were not receiving immunosuppressive medications had increasing Th17/Treg ratios, whereas patients who were receiving immunosuppressive medications had decreasing Th17/Treg ratios (*p* = 0.006 for difference) (Fig. 6g). The trajectories of Th1/Treg and Th2/Treg were not significantly different for survivors among patients who were and those who were not receiving immunosuppressive medications (Fig. 6h, i).

## Discussion

In this study of immune responses to *S. aureus* bloodstream infections, we found that two immune marker ratios were associated with increased risk of death. Interestingly, high neutrophil counts and Th17 cytokine responses were not associated with survival in isolation. Rather, neutrophil counts and Th17 cytokine responses were associated with survival only when they were high relative to lymphocyte counts and Th1 cytokine responses, respectively. The average values for these immune marker ratios were similar for patients with and without immunocompromising conditions, owing to the fact that the presence of an immunocompromising condition was associated with proportionately lower neutrophil counts, lymphocyte counts, Th17 cytokine scores, and Th1 cytokine scores.

Patients who are immunocompromised because they have hematopoietic conditions or because they require treatment with immunosuppressive medications are typically viewed as having increased risk of death from sepsis. However, patients with these conditions in our cohort did not have increased 90-day mortality, confirming the results of a prior chart review [23] . The reason for this finding may be that immunocompromising conditions may have both beneficial and harmful effects on the host’s immune response to *S. aureus*. For instance, such a condition may attenuate a harmful proinflammatory neutrophil or Th17 response. However, being immunocompromised was associated with a lower lymphocyte and Th1 response, perhaps compromising the ability of the patient to clear the infection or increasing the risk of a subsequent infection.

A strength of our study is that we avoided introducing heterogeneity with respect to causative pathogens. We included only patients with a definite infection due to a single pathogen that typically causes systemic inflammation and severe acute illness that can result in the development of sepsis. Because sepsis is defined by a clinical response to a presumed infection, it is difficult to identify prognostic immune biomarkers when the pathogens in a study are heterogeneous and some patients may not even be infected [4]. In fact, prior investigators have shown that the type of immune dysregulation in sepsis depends on the type of pathogen [36, 37].

Th17 responses have been considered protective during staphylococcal infections, based largely on preclinical mouse studies, as well as the observation that patients with inborn errors in Th17 cells are prone to invasive staphylococcal infections [19]. Although our findings that Th17 responses were associated with increased mortality may seem counterintuitive, we note that patients who received the Merck V710 staphylococcal vaccine had markedly *increased* mortality due to *S. aureus* infection compared with placebo recipients, suggesting that the vaccination strategy generated a lethal immune response [38]. In light of our data, we hypothesize that the optimal Th17 response must be adequate to clear pathogen but not so overwhelming as to precipitate inflammatory damage and death to the host. In addition, the Th17 response must be considered relative to other elements of the adaptive immune response, including Th1, Th2, and Treg responses, all of which may be protective.

The observation that Th2 responses were more likely to be elevated among survivors than non-survivors is interesting, especially in light of recent work indicating an association between type 2/Th2 diseases (such as asthma and allergy) and development of sepsis or sepsis outcomes [39, 40]. These epidemiologic studies suggest that the presence of allergic diseases or asthma reduces the risk for developing sepsis as well as sepsis-associated mortality. We do not have information about allergic comorbidities among these patients. However, given that Th2 responses evolved to facilitate tissue repair and can attenuate proinflammatory Th17 or Th1 responses, the presence of a preexisting Th2 disease may facilitate the resolution of the inflammatory insult.

Tregs provide a crucial check on the early proinflammatory response, but their presence is thought to contribute to the vulnerability of patients to subsequent infections in the later phase of sepsis. When Treg percentage was analyzed using multivariable analysis, adjusting for Th2 and Th17 responses, a high early Treg percentage was associated with increased mortality. During the course of the infection, an increasing Treg response relative to Th17 response was associated with decreased mortality. These results indicate that consideration of all elements of the adaptive immune response for a given infection provides a more accurate indication of the immune dysregulation of sepsis associated with death.

Our study has several limitations: First, our study was limited by its small size. The cytokine aspect of our study was powered to include two variables in a Cox proportional hazards model. We did not perform a sample size calculation prior to our flow cytometry experiment, because we explored infrequently studied immune markers in a group of patients that is typically excluded from sepsis biomarker research. Second, there is no universally accepted definition of an immunocompromised patient [9, 41–43]. Because there is a wide range of types and degrees of severity of immune impairment among patients who are commonly classified as immunocompromised, we categorized patients who had hematopoietic disorders separately from those who were taking immune-altering medications. Third, our evaluation of how immunocompromising conditions affect the immune response to *S. aureus* requires confirmation in future adequately powered studies. There were fewer than 10 patients with hematologic malignancies or HIV infections, and none of them died within 90 days of infection. As a result, we did not include any of these patients in the flow cytometry portion of our study. Finally, although we studied infections of the same pathogen type, there were likely genetic differences among *S. aureus* organisms that we did not account for and that could have affected the immune response and risk of death. The majority of the *S. aureus* infections in our study were community-acquired and methicillin-sensitive; neither of these characteristics was associated with mortality.

## Conclusions

We found that among patients with *S. aureus* bacteremia, both increased neutrophil-to-lymphocyte count ratios and increased Th17 immune responses relative to Th1 responses were most strongly associated with increased mortality. Our data suggest that medical conditions and medications that directly affect the immune system may protect patients from a strong Th17 immune response. Conversely, a weak Th1 immune response may identify patients with immunocompromised states who have persistent vulnerability to recurrent infections [44]. One might speculate that anti-IL-6 therapy may be beneficial only for a subgroup of patients with normal immune states who also have an elevated Th17 level. In contrast, patients with immunocompromising conditions may benefit from interferon-γ therapy to boost Th1 immunity during infection. Future strategies to develop immune-modulatory therapies for sepsis will require personalization to the immune state of the patient and the associated dysregulated immune response.

## Additional files

Additional file 1: **Figure S1.** Enrollment. Between July 1, 2013, and October 24, 2014, there were weeks when no patient was considered for enrollment, during which 33 patients had *S. aureus* bacteremia. (PPTX 3105 kb)

Additional file 2: **Table S1.** Associations between each cytokine level at time point 1 and death during the first 90 days after infection. **Table S2.** Associations between each cytokine level at time point 2 and death during the first 90 days after infection. **Table S3.** Method used to calculate Th17, Th1, and Th2 scores. **Table S4.** Associations between helper T cell scores at time point 2 and death during the first 90 days after *S. aureus* bacteremia. **Table S5.** Associations between the change in helper T cell scores per day and death during the first 90 days after *S. aureus* bacteremia. **Table S6.** Baseline characteristics of 95 patients with *S. aureus* bacteremia. **Table S7.** Comparison of clinical characteristics of 28 patients included (columns 1 and 2) and 67 patients not included (columns 3 and 4) in the flow cytometric analysis. **Table S8.** Cellular markers early after infection and over the course of the infection among 14 patients who died and 14 patients who survived. (DOCX 50 kb)

Additional file 3: **Figure S2.** Method used to classify T cells and CD3^+^CD4^+^ cells. CD4^+^ T cells were first separated into CD25^+^CD127^−^ (Tregs) or conventional T cells (Tconv). The Tconv were further characterized by expression of CD45RO as a marker of effector/memory T cells. The CD45RO^+^ cells were then divided into Th1, Th2, or Th17 cells based on the expression of CCR4 and CCR6, with Th1 identified as CCR4^−^CCR6^−^, Th2 identified as CCR4^+^CCR6^−^, and Th17 identified as CCR6^+^. (PPTX 84 kb)

Additional file 4: **Figure S3** Individual patient trajectories for T-cell subsets. T-cell subset percentages over time for each individual patient are shown, with survivors (at 90 days) indicated by a solid line and non-survivors indicated by a dashed line. (PPTX 670 kb)

Additional file 5: **Figure S4** Method for determining a patient’s T-cell subset levels. Linear regression was used to predict the change in each T-cell subset percentage per day for each patient. Each regression line was used to estimate a T-cell percentage at day 3 after infection. The day 3 value and change over time were used as predictor variables in Cox proportional hazards models. (PPTX 2104 kb)

## Abbreviations

APC: Allophycocyanin
CARS: Compensatory anti-inflammatory response syndrome
Cy7: Cyanine 7
HIV: Human immunodeficiency virus
HLA: Human leukocyte antigen
IL: Interleukin
PBMCs: Peripheral blood mononuclear cells
PE: Phycoerythrin
SIRS: Systemic inflammatory response syndrome
SOFA: Sequential Organ Failure Assessment
Th: T-helper cell
TNF: Tumor necrosis factor
Treg: Regulatory T cell
WBC: White blood cells
