# Supplementary figures and images for: Distinct T-helper cell responses to Staphylococcus aureus bacteremia reflect immunologic comorbidities and correlate with mortality
Source: Crit Care. 2018 Apr 25;22:107. doi: 10.1186/s13054-018-2025-x (PMC5916828; doi:10.1186/s13054-018-2025-x)

## Slide 1
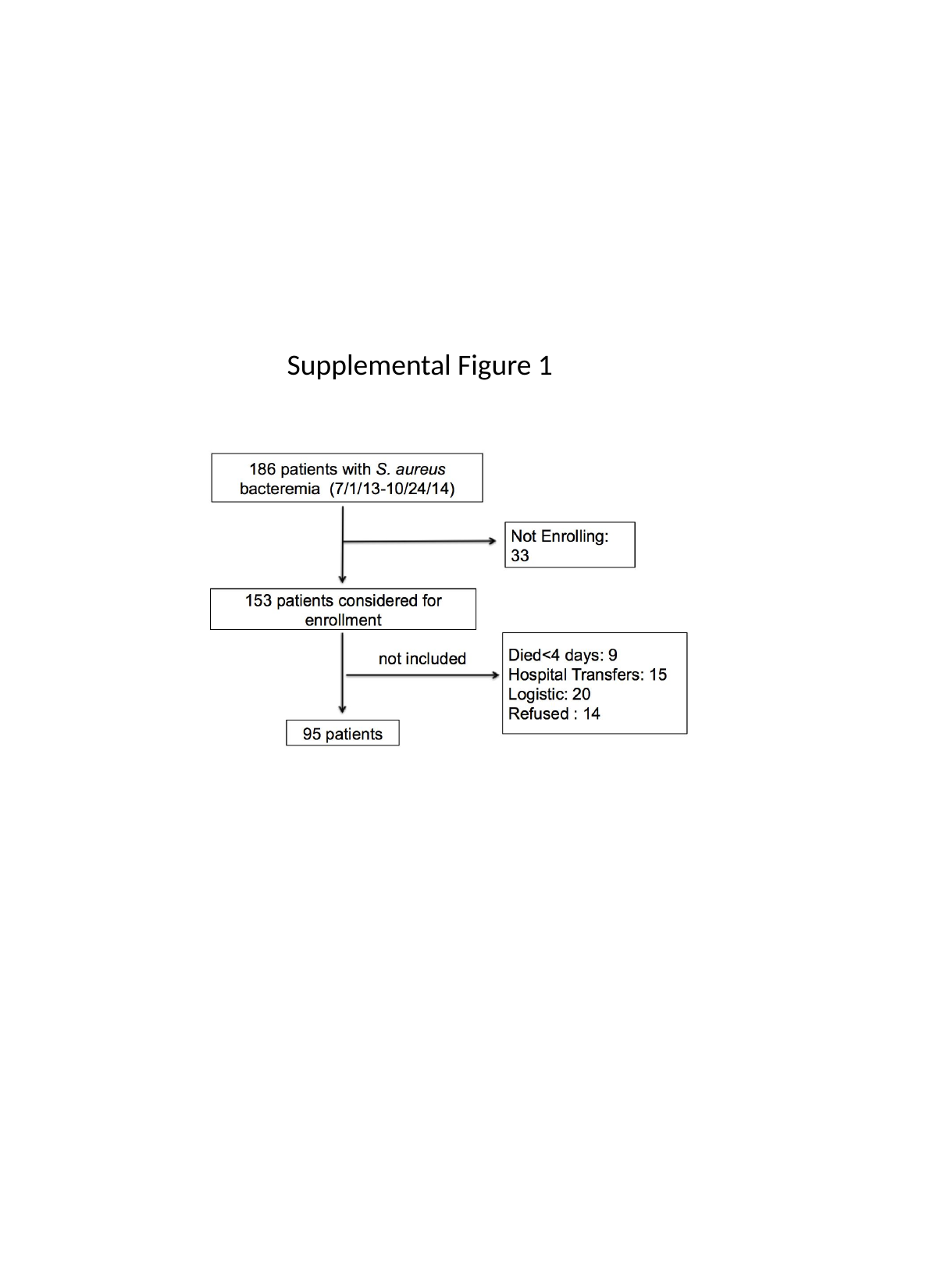

Supplemental Figure 1

Supplement: Supplementary file 1 — Figure S1. Enrollment. Between July 1, 2013, and October 24, 2014, there were weeks when no patient was considered for enrollment, during which 33 patients had S. aureus bacteremia. (PPTX 3105 kb) [file 13054_2018_2025_MOESM1_ESM.pptx]

## Slide 1
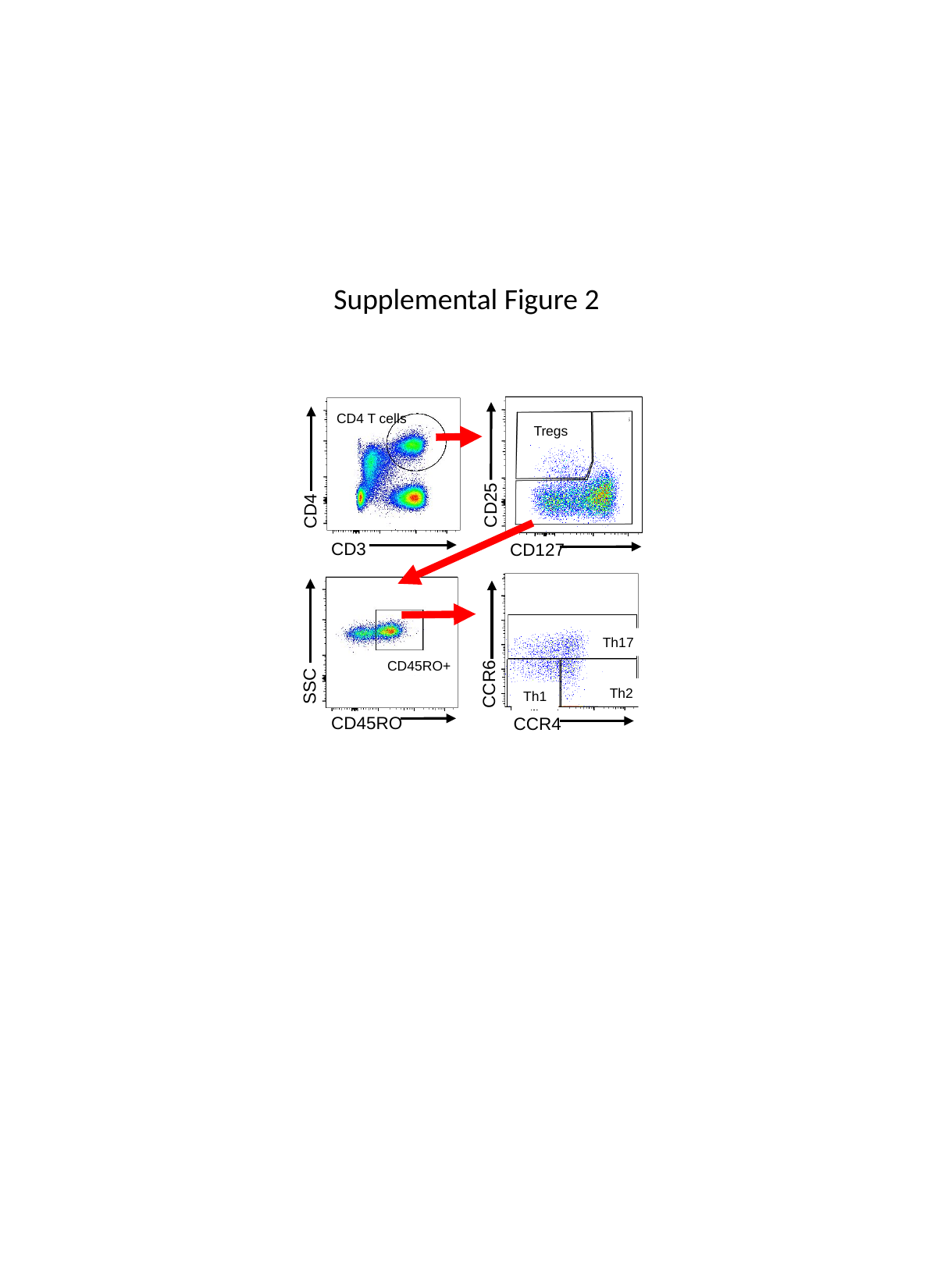

Supplemental Figure 2
CD4
CD4 T cells
Tregs
CD4
SSC
CD25
CD3
CD127
Th17
CD45RO+
CCR6
Th2
Th1
CD45RO
CCR4

Supplement: Supplementary file 3 — Figure S2. Method used to classify T cells and CD3+CD4+ cells. CD4+ T cells were first separated into CD25+CD127− (Tregs) or conventional T cells (Tconv). The Tconv were further characterized by expression of CD45RO as a marker of effector/memory T cells. The CD45RO+ cells were then divided into Th1, Th2, or Th17 cells based on the expression of CCR4 and CCR6, with Th1 identified as CCR4−CCR6−, Th2 identified as CCR4+CCR6−, and Th17 identified as CCR6+. (PPTX 84 kb) [file 13054_2018_2025_MOESM3_ESM.pptx]

## Slide 1
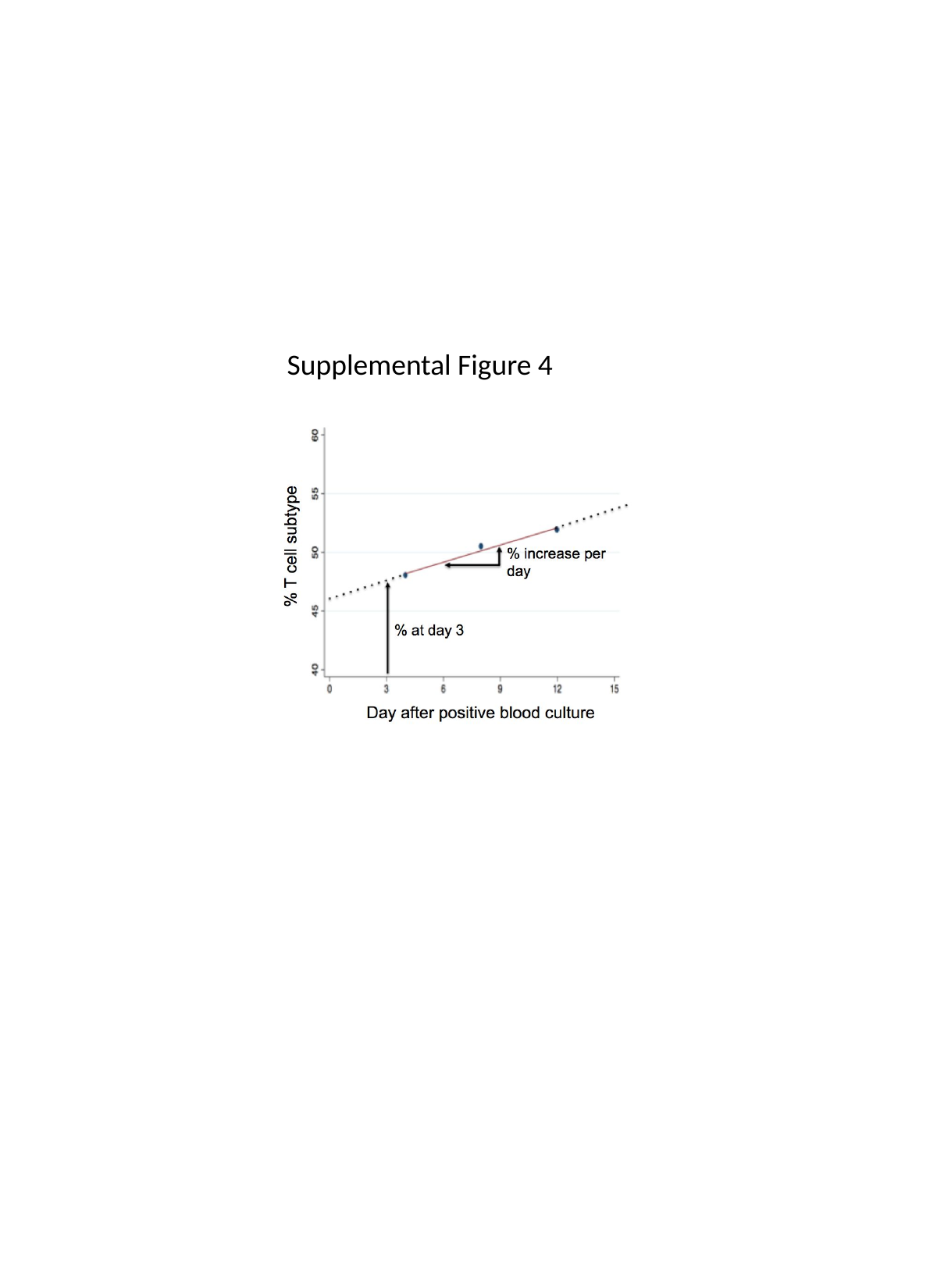

Supplemental Figure 4

Supplement: Supplementary file 5 — Figure S4 Method for determining a patient’s T-cell subset levels. Linear regression was used to predict the change in each T-cell subset percentage per day for each patient. Each regression line was used to estimate a T-cell percentage at day 3 after infection. The day 3 value and change over time were used as predictor variables in Cox proportional hazards models. (PPTX 2104 kb) [file 13054_2018_2025_MOESM5_ESM.pptx]
