# Supplementary material for: Distinct T-helper cell responses to Staphylococcus aureus bacteremia reflect immunologic comorbidities and correlate with mortality
Source: Crit Care. 2018 Apr 25;22:107. doi: 10.1186/s13054-018-2025-x (PMC5916828; doi:10.1186/s13054-018-2025-x)
Supplement: Supplementary file 2 — Table S1. Associations between each cytokine level at time point 1 and death during the first 90 days after infection. Table S2. Associations between each cytokine level at time point 2 and death during the first 90 days after infection. Table S3. Method used to calculate Th17, Th1, and Th2 scores. Table S4. Associations between helper T cell scores at time point 2 and death during the first 90 days after S. aureus bacteremia. Table S5. Associations between the change in helper T cell scores per day and death during the first 90 days after S. aureus bacteremia. Table S6. Baseline characteristics of 95 patients with S. aureus bacteremia. Table S7. Comparison of clinical characteristics of 28 patients included (columns 1 and 2) and 67 patients not included (columns 3 and 4) in the flow cytometric analysis. Table S8. Cellular markers early after infection and over the course of the infection among 14 patients who died and 14 patients who survived. (DOCX 50 kb) [file 13054_2018_2025_MOESM2_ESM.docx]

**Table S1** – *Associations between each cytokine level at time point 1 and death during the first 90 days after infection.*  The association between each cellular marker and death was determined using a Cox proportional hazard model.

| Pathway | Cytokines | Percent of Patients Expressing Cytokine | Associations between each variable and death during the 90 days after infection | | |
| --- | --- | --- | --- | --- | --- |
|  |  |  | HR | 95% CI | p value |
| Th17 | IL-6 pg/mL | 96 | 1.72 | 1.25-2.37 | <0.01 |
|  | IL-17A pg/mL | 11 | 2.36 | 1.35-4.15 | <0.01 |
|  | IL-17F ng/mL | 55 | 1.18 | 0.93-1.50 | 0.18 |
|  | IL-21 pg/mL | 68 | 0.87 | 0.72-1.06 | 0.17 |
|  | IL-22 ng/mL | 49 | 1.41 | 0.92-2.15 | 0.11 |
|  | IL-23 ng/mL | 57 | 0.98 | 0.83-1.14 | 0.76 |
| Th1 | IL-2 pg/mL | 19 | 1.00 | 0.85-1.19 | 0.96 |
|  | IFNγ pg/mL | 40 | 0.86 | 0.60-1.24 | 0.42 |
|  | IL-12 pg/mL | 64 | 0.88 | 0.71-1.08 | 0.23 |
|  | TNFα pg/mL | 100 | 1.56 | 0.86-2.84 | 0.15 |
| Th2 | IL-4 ng/mL | 23 | 1.08 | 0.57-2.05 | 0.82 |
|  | IL-5 pg/mL | 19 | 0.91 | 0.54-1.51 | 0.71 |
|  | IL-9 pg/mL | 23 | 1.13 | 0.46-0.82 | 0.46 |
|  | IL-13 pg/mL | 36 | 1.06 | 0.76-1.47 | 0.74 |
|  | IL-25 ng/mL | 30 | 1.16 | 0.91-1.47 | 0.23 |
|  | IL-31 ng/mL | 60 | 0.99 | 0.75-1.30 | 0.93 |
|  | IL-33 pg/mL | 77 | 0.91 | 0.67-1.23 | 0.55 |
| Uncategorized | IL-10 pg/mL | 82 | 1.31 | 0.97-1.77 | 0.08 |

**Table S2** – *Associations between each cytokine level at time point 2 and death during the first 90 days after infection.*  The association between each cellular marker and death was determined using a Cox proportional hazard model.

| Pathway | Cytokines | Percent of Patients Expressing Cytokine | Associations between each variable and death during the 90 days after infection | | |
| --- | --- | --- | --- | --- | --- |
|  |  |  | HR | 95% CI | p value |
| Th17 | IL-6 pg/mL | 99 | 2.03 | 1.31-3.14 | 0.002 |
|  | IL-17A pg/mL | 8 | 1.21 | 0.31-4.72 | 0.78 |
|  | IL-17F ng/mL | 70 | 1.32 | 0.98-1.78 | 0.07 |
|  | IL-21 pg/mL | 58 | 0.91 | 0.72-1.16 | 0.46 |
|  | IL-22 ng/mL | 74 | 1.68 | 0.93-3.05 | 0.09 |
|  | IL-23 ng/mL | 44 | 1.04 | 0.82-1.31 | 0.76 |
| Th1 | IL-2 pg/mL | 59 | 1.01 | 0.68-1.50 | 0.97 |
|  | IFNγ pg/mL | 63 | 0.96 | 0.72-1.29 | 0.8 |
|  | IL-12 pg/mL | 47 | 0.99 | 0.80-1.22 | 0.92 |
|  | TNFα pg/mL | 99 | 1.41 | 0.67-3.00 | 0.37 |
| Th2 | IL-4 ng/mL | 21 | 1.31 | 0.66-2.61 | 0.44 |
|  | IL-5 pg/mL | 58 | 0.84 | 0.52-1.38 | 0.5 |
|  | IL-9 pg/mL | 26 | 1.09 | 0.75-1.59 | 0.66 |
|  | IL-13 pg/mL | 63 | 1.2 | 0.84-1.72 | 0.32 |
|  | IL-25 ng/mL | 15 | 1.16 | 0.87-1.55 | 0.31 |
|  | IL-31 ng/mL | 45 | 1.01 | 0.73-1.41 | 0.94 |
|  | IL-33 pg/mL | 77 | 0.99 | 0.73-1.35 | 0.96 |
| Uncategorized | IL-10 pg/mL | 78 | 1.16 | 0.84-1.60 | 0.37 |

**Table S3** – *Method to calculate Th17, Th1, and Th2 scores*. Each cytokine level was standardized by dividing its measured value by the cohort’s median value. Each patient’s Th17, Th1, and Th2 score was determined by summing all log standardized cytokine concentrations from the same immune pathway.

| Pathway | Cytokines | Score for each patient "i" |
| --- | --- | --- |
| Th17 | IL-6 | log(IL-6_i_/43.49) + log(IL-17A_i_/0.02) + log(IL-17F_i_/0.87) + log(IL-21_i_/0.61) + log(IL-22_i_/0.34) + log(IL-23_i_/0.01) |
|  | IL-17A |  |
|  | IL-17F |  |
|  | IL-21 |  |
|  | IL-22 |  |
|  | IL-23 |  |
| Th1 | IL-2 | log(IL-2_i_/1.45) + log(IFNγ_i_/0.92) + log(IL-12_i_/0.06) + log(TNFα_i_/25.1) |
|  | IFNγ |  |
|  | IL-12 |  |
|  | TNFα |  |
| Th2 | IL-4 | log(IL-4_i_/0.02) + log(IL-5_i_/0.46) + log(IL-9_i_/0.73) + log(IL-13_i_/4.44) + log(IL-25_i_/0.01) + log IL-31_i_/0.01 + log(IL33_i_/3.2) |
|  | IL-5 |  |
|  | IL-9 |  |
|  | IL-13 |  |
|  | IL-25 |  |
|  | IL-31 |  |
|  | IL-33 |  |

**Table S4** – *Associations between helper T cell scores at time point 2 and death during 90 days after S. aureus bacteremia*. Associations between each helper T cell score and death was determined using a Cox proportional hazard model. Three multivariable Cox proportional hazard models were performed, each including two of the three helper T cell scores as predictor variables. A “-“ signifies a variable that was not included in the model. A total of 71 of 95 patients (75%) had cytokines measured at time point 2.

|  | Associations between each variable and death during the 90 days after infection | | | Multivariable model of Th17 and Th1 scores | | | Multivariable model of Th17 and Th2 scores | | | Multivariable model of Th1 and Th2 scores | | |
| --- | --- | --- | --- | --- | --- | --- | --- | --- | --- | --- | --- | --- |
|  |  |  |  |  |  |  |  |  |  |  |  |  |
|  | HR | 95% CI | p value | HR | 95% CI | p value | HR | 95% CI | p value | HR | 95% CI | p value |
| Th17 score at time point 2 | 1.03 | 0.96-1.12 | 0.3 | 1.12 | 0.99-1.27 | 0.08 | 1.13 | 0.96-1.33 | 0.15 | - | - | - |
| Th1 score at time point 2 | 1.00 | 0.91-1.11 | 1.00 | 0.89 | 0.75-1.05 | 0.18 | - | - | - | 0.95 | 0.78-1.15 | 0.57 |
| Th2 score at time point 2 | 1.01 | 0.95-1.08 | 0.69 | - | - | - | 0.92 | 0.79-1.07 | 0.30 | 1.05 | 0.92-1.19 | 0.49 |

**Table S5 -** *Associations between the change in helper T cell scores per day and death during 90 days after S. aureus bacteremia*. Associations between each change in helper T cell score per day and death was determined using a Cox proportional hazard model. Three multivariable Cox proportional hazard models were performed, each including two of the three helper T cell scores as predictor variables. A “-“ signifies a variable that was not included in the model.

|  | Associations between each variable and death during the 90 days after infection | | | Multivariable model of Th17 and Th1 scores | | | Multivariable model of Th17 and Th2 scores | | | Multivariable model of Th1 and Th2 scores | | |
| --- | --- | --- | --- | --- | --- | --- | --- | --- | --- | --- | --- | --- |
|  |  |  |  |  |  |  |  |  |  |  |  |  |
|  | HR | 95% CI | p value | HR | 95% CI | p value | HR | 95% CI | p value | HR | 95% CI | p value |
| Δ Th17 score per day | 1.05 | 0.68-1.61 | 0.84 | 1.06 | 0.53-2.10 | 0.88 | 1.02 | 0.51-2.05 | 0.96 | - | - | - |
| Δ Th1 score per day | 1.04 | 0.59-1.84 | 0.90 | 0.98 | 0.39-2.45 | 0.97 | - | - | - | 0.98 | 0.42-2.30 | 0.97 |
| Δ Th2 score per day | 1.04 | 0.71-1.53 | 0.83 | - | - | - | 1.03 | 0.55-1.91 | 0.93 | 1.05 | 0.59-1.86 | 0.86 |

**Table S6 -** *Baseline characteristics of 95 patients with S. aureus bacteremia*. Patient were grouped as having an immunosuppressive medical condition or receiving an immunosuppressive medication or not.

|  | | Immunosuppressive Medical Condition or Medication | No Immunosuppressive Medical Condition or Medication | p value |
| --- | --- | --- | --- | --- |
|  |  | N=44 | N=51 |  |
| Patient Background | Years of age, mean [SD] | 58 [15] | 58 [16] | 0.86 |
|  | Male, n (%) | 23 (52) | 35 (69) | 0.10 |
|  | Black, n (%) | 20 (45) | 39 (76) | 0.002 |
|  | Diabetes, n (%) | 11 (25) | 23 (45) | 0.04 |
|  | Congestive heart failure, n (%) | 4 (9) | 25 (49) | <0.001 |
|  | End-stage renal disease, n (%) | 7 (16) | 17 (33) | 0.06 |
|  | Coronary artery disease, n (%) | 3 (7) | 18 (35) | 0.001 |
| Clinical response on first day of infection | Positive SIRS Temperature criteria, n (%) | 34 (77) | 35 (69) | 0.35 |
|  | Positive SIRS Heart Rate criteria. n (%) | 38 (86) | 34 (67) | 0.03 |
|  | Positive SIRS Respiratory criteria, n (%) | 39 (89) | 47 (92) | 0.56 |
|  | Positive SIRS Temperature criteria WBC criteria N (%) | 33 (75) | 37 (73) | 0.79 |
|  | SOFA score on first day of positive blood cultures, mean [SD] | 4 [4] | 4 [2] | 0.7 |
|  | Median time to antibiotics, h (IQR) | 2.2 (1.0 – 6.0) | 2.1 (0.5 – 8.6) | 0.89 |
| Characteristics of Infection | Methicillin Resistance, n (%) | 16 (36) | 17 (33) | 0.76 |
|  | Positive cultures <48hrs from admission, n (%) | 36 (81) | 46 (92) | 0.14 |
|  | Days of Positive Cultures, mean [SD] | 2 [1, 4] | 2 [1, 3] | 0.74 |
|  | Endocarditis, n (%) | 4 [9] | 6 [12] | 0.68 |
|  | Removable Source*, n (%) | 19 (43) | 13 (25) | 0.07 |
|  | Skin/Soft Tissue, n (%) | 11 (25) | 19 (37) | 0.2 |
|  | Pulmonary, n (%) | 3 (6.8) | 9 (18) | 0.13 |
|  | Other Source, n (%) | 1 (2) | 1 (2) | 1 |
|  | Undetermined Source, n (%) | 10 (23) | 9 (18) | 0.54 |

**Table S7 -** Comparison of clinical characteristics for 28 patients included (columns 1 and 2) and 67 patients not included (columns 3 and 4) in the flow cytometry aspect of the study.

|  | Included and Died | Included and Survived | Not Included and Died | Not Included and Survived | p value for column 1 vs column 2 | p value for column 1 vs column 3 | p value for column 2 vs column 4 |
| --- | --- | --- | --- | --- | --- | --- | --- |
|  | N=14 | N=14 | N=7 | N=60 |  |  |  |
| Years of age, mean [SD] | 63 [11] | 54 [14] | 69 [16] | 56 [16] | 0.1 | 0.34 | 0.66 |
| Male, n (%) | 8 (57) | 9 (64) | 4 (57) | 37 (62) | 1 | 1 | 1 |
| Black, n (%) | 9 (64) | 9 (64) | 3 (43) | 38 (63) | 1 | 0.4 | 1 |
| Immunosuppressive medical condition, n (%) | 7 (50) | 7 (50) | 4 (57) | 17 (28) | 1 | 1 | 0.2 |
| Immunosuppressive medication, n (%) | 0 (0) | 0 (0) | 0 (0) | 9 (15) | n/a | n/a | 0.19 |
| No Immunosuppressive medical condition or medication, n (%) | 7 (50) | 7 (50) | 3 (43) | 34 (57) | 1 | 1 | 0.77 |
| Neutrophils (K cells/µL) on first day of bacteremia, mean [SD] | 13 [6] | 9.7 [6] | 16 [12] | 11 [6] | 0.13 | 0.45 | 0.50 |
| Lymphocytes (K cells/µL) on first day of bacteremia, mean [SD] | 0.6 [0.4] | 0.8 [0.6] | 0.9 [1.2] | 1.1 [0.8] | 0.48 | 0.92 | 0.62 |
| Th17 cytokine score at time point 1 | 4.5 [7.8] | -0.81 [4.3] | 5.2 [9.1] | 3.1 [6.3] | 0.04 | 0.85 | 0.03 |
| Th1 cytokine score at time point 1 | 1.8 [4.3] | 0.1 [3.1] | 0.8 [5.4] | 2.3 [5.0] | 0.23 | 0.65 | 0.12 |
| Th2 cytokine score at time point 1 | 3.5 [8.7] | -1.2 [1.9] | 2.5 [9.1] | 3.2 [6.9] | 0.06 | 0.83 | 0.02 |
| Methicillin Resistance, n (%) | 3 (21) | 5 (36) | 2 (29) | 23 (38) | 0.68 | 1 | 1 |
| Days of positive cultures, mean [SD] | 3 [2] | 5 [4] | 4 [3] | 3 [2] | 0.1 | 0.4 | 0.02 |
| SOFA score, mean [SD] | 5 [3] | 4 [2] | 10 [6] | 3 [2] | 0.74 | 0.01 | 0.11 |

**Table S8** – *Cellular markers early after infection and over the course of the infection among 14 patients who died and 14 patients who survived*.

|  | Average among patients who died (SD) | Average among patients who survived (SD) | p value for difference |
| --- | --- | --- | --- |
| Th17 at day 3 | 52 (17) | 47 (11) | 0.37 |
| Th1 at day 3 | 13 (6) | 13 (6) | 0.97 |
| Th2 at day 3 | 26 (8) | 36 (9) | <0.01 |
| Treg at day 3 | 10 (6) | 7 (4) | 0.09 |
| Th17 / Treg at day 3 | 6 (4) | 10 (8) | 0.16 |
| Th1 / Treg at day 3 | 2 (1) | 3 (2) | 0.10 |
| Th2 / Treg at day 3 | 3 (2) | 8 (8) | 0.04 |
| ΔTh17 / day | 1.3 (1.7) | 0.3 (1.3) | 0.10 |
| ΔTh1 / day | -0.6 (0.2) | -0.1 (0.3) | 0.06 |
| ΔTh2 / day | -0.2 (1.8) | -0.1 (1.1) | 0.89 |
| ΔTreg / day | -0.4 (0.9) | -0.1 (0.6) | 0.26 |
| Δ(Th17 / Treg) / day | 0.55 (1.1) | 0.05 (0.4) | 0.12 |
| Δ(Th1 / Treg) / day | -0.01 (0.2) | -0.04 (0.1) | 0.63 |
| Δ(Th2 / Treg) / day | 0.06 (0.4) | -0.06 (0.3) | 0.36 |
