# Supplementary material for: Distinct T-helper cell responses to Staphylococcus aureus bacteremia reflect immunologic comorbidities and correlate with mortality
Source: Crit Care. 2018 Apr 25;22:107. doi: 10.1186/s13054-018-2025-x (PMC5916828; doi:10.1186/s13054-018-2025-x)
Supplement: Supplementary file 4 — Figure S3 Individual patient trajectories for T-cell subsets. T-cell subset percentages over time for each individual patient are shown, with survivors (at 90 days) indicated by a solid line and non-survivors indicated by a dashed line. (PPTX 670 kb) [file 13054_2018_2025_MOESM4_ESM.pptx]

## Slide 1
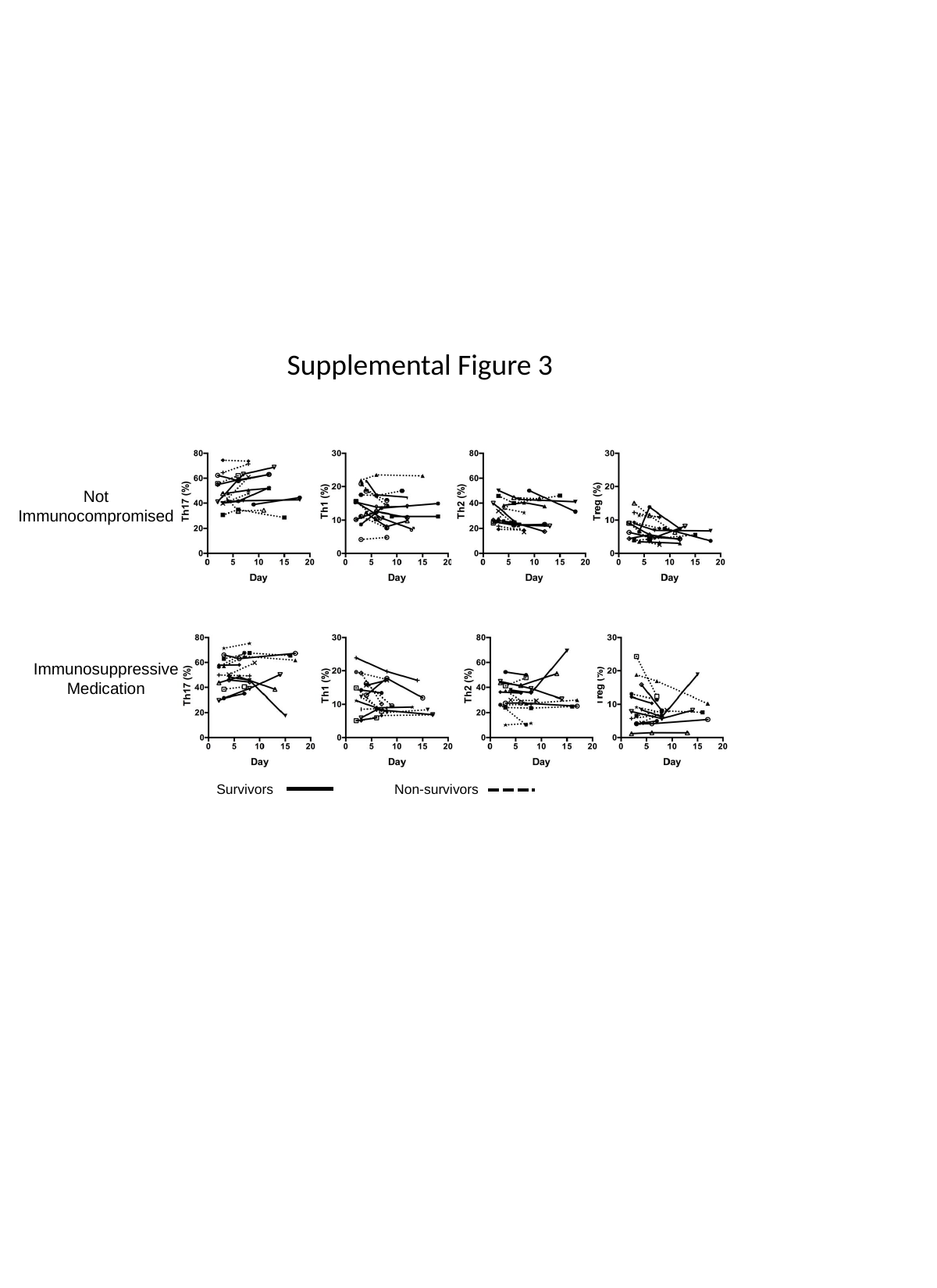

Supplemental Figure 3
Survivors
Non-survivors
Not Immunocompromised
Immunosuppressive Medication
